# Odontoid fracture in geriatric patients — analysis of complications and outcome following conservative treatment vs. ventral and dorsal surgery

**DOI:** 10.1186/s12877-023-04472-2

**Published:** 2023-11-15

**Authors:** Matthias K. Jung, Lukas Hörnig, Philipp Raisch, Paul A. Grützner, Michael Kreinest

**Affiliations:** Department of Trauma and Orthopaedic Surgery, BG Klinik Ludwigshafen, Ludwigshafen, Germany

**Keywords:** Odontoid fracture, Geriatric patient, Conservative treatment, Ventral screw osteosynthesis, Dorsal instrumentation

## Abstract

**Background:**

Different treatment options are discussed for geriatric odontoid fracture. The aim of this study was to compare the treatment options for geriatric odontoid fractures.

**Methods:**

Included were patients with the following criteria: age ≥ 65 years, identification of seniors at risk (ISAR score ≥ 2), and odontoid fracture type A/B according to Eysel and Roosen. Three groups were compared: conservative treatment, surgical therapy with ventral screw osteosynthesis or dorsal instrumentation. At a follow-up examination, the range of motion and the trabecular bone fracture healing rate were evaluated. Furthermore, demographic patient data, neurological status, length of stay at the hospital and at the intensive care unit (ICU) as well as the duration of surgery and occurring complications were analyzed.

**Results:**

A total of 72 patients were included and 43 patients could be re-examined (range: 2.7 ± 2.1 months). Patients with dorsal instrumentation had a better rotation. Other directions of motion were not significantly different. The trabecular bone fracture healing rate was 78.6%. The patients with dorsal instrumentation were hospitalized significantly longer; however, their duration at the ICU was shortest. There was no significant difference in complications.

**Conclusion:**

Geriatric patients with odontoid fracture require individual treatment planning. Dorsal instrumentation may offer some advantages.

## Background

Due to demographic changes, the number of geriatric trauma patients is constantly increasing [1]. Geriatric patients, having a worse general condition and a corresponding increased need for extensive care, can be identified by using the seniors at risk (ISAR) score [2–4] upon admission to the hospital. In these geriatric patients, odontoid fractures are the most common, comprising more than 50% of fractures of the cervical spine [5].

In contrast to odontoid fractures in young patients, these injuries in the geriatric population are mainly caused by minor trauma and falls at home [6]. The poor bone quality of the geriatric population together with pre-existing conditions such as osteopenia and osteoporosis are one of the main reasons for occurrence of this fracture [7].

**Fig. 1:**
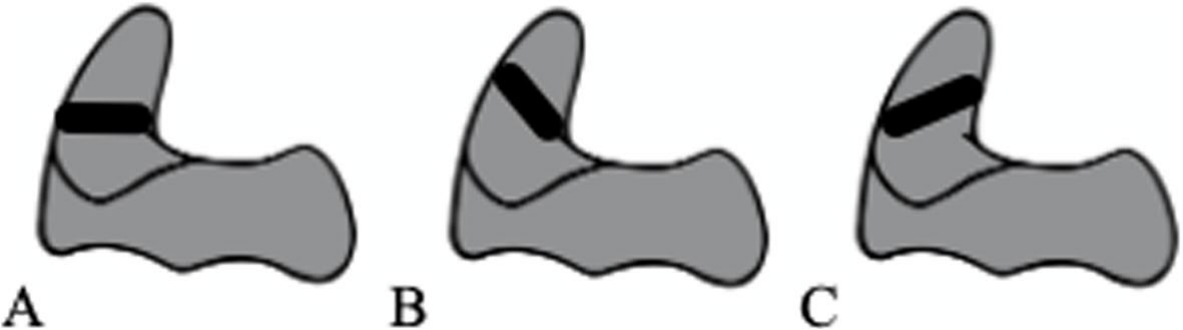
Odontoid fracture according to Eysel and Roosen; type A: horizontal fracture line (**A**); type B ventrocranial to dorsocaudal fracture line (**B**); type C ventrocaudal to dorsocranial fracture line (**C**)

In 1974, Anderson and D’Alonzo established a classification system for odontoid fractures according to fracture morphology [8]: type I fractures affect the tip of the odontoid, type II fractures affect the base of the odontoid, and in type III fractures, the corpus of the axis is affected. Around 19 years later, type II odontoid fractures according to Anderson and D’Alonzo were subclassified by Eysel and Roosen for association with different treatment options (Fig. 1): type A is a horizontal fracture (Fig. 1A) that can be treated with ventral screw osteosynthesis; type B is a fracture line from ventrocranial to dorsocaudal (Fig. 1B) that can be treated like type A; type C is a fracture line from ventrocaudal to dorsocranial (Fig. 1C) that can be treated with dorsal instrumentation of C1 and C2 [9–11]. Whereas the ventral screw osteosynthesis could only be applied in type A and type B fractures due to an increased risk for a ventral dislocation of the odontoid, the dorsal instrumentation, may be applicable in all fracture types according to Eysel and Roosen [12].

Surgical treatment by different surgical approaches as well as conservative treatment may be associated with various complications and high mortality rates, especially in patients with reduced general condition [13–20].

The aim of the present study was to compare clinical and radiological outcome as well as peri-operative data and complications for geriatric patients with type A and B odontoid fractures according to Eysel and Roosen following conservative treatment vs. operative treatment by ventral vs. dorsal stabilization.

## Methods

The present study was approved by the local ethics commission in charge (Ethics Committee of the State Medical Association Rhineland-Palatinate, Mainz, Germany). All patients who met the following criteria were included in this retrospective single-center cohort study: (i) age of 65 years or older, (ii) ISAR [2, 3] score of 2 or higher, (iii) fracture of the odontoid type A or B according to Eysel and Roosen [9], and (iv) treatment between January 2012 and December 2017. To obtain a homogeneous patient population, we excluded non-geriatric patients as well as patients suffering from other C2 fractures.

### Clinical evaluation

The demographic data as well as the prehospital and treatment data of the patients were documented as a standard procedure. Data on pre-existing conditions, trauma mechanism, Glasgow Coma Scale (GCS), ISAR score and neurological status were collected for all patients. The ISAR score can be used to assess the need for assistance among geriatric patients. From a score of 2, there is a significant need for additional assistance for the geriatric patients [2–4]. The duration of hospital stay, the duration of stay at the intensive care unit (ICU), and the duration of surgery were registered.

### Conservative and surgical treatment

All patients underwent a clinical and neurological examination as well as a computer tomography (CT) scan of the cervical spine on the day of admission to the hospital. Further treatment was mainly based on the recommendations given by Eysel and Roosen [9]. However, the decision on treatment was influenced by dislocation and instability [10] of the odontoid fracture. With increasing instability and dislocation, dorsal instrumentation was performed. Dorsal surgical therapy was performed especially in cases of dislocation of more than two millimeters. Otherwise, ventral screw osteosynthesis was performed. Conservative treatment was performed on patients in whom there was no dislocation.

#### Conservative treatment

The cervical spine was immobilized for 6 weeks in a soft or rigid collar. Regular clinical controls were performed to evaluate the skin under the collar and the patient’s condition.

#### Surgical treatment

**Fig. 2:**
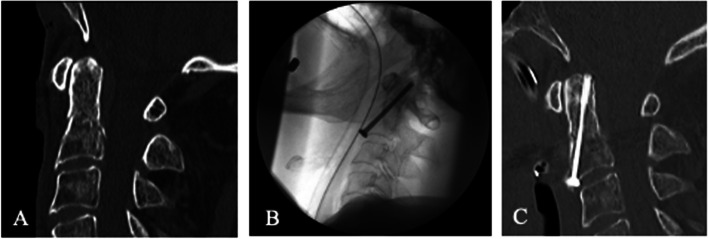
An 92–year–old male patient with odontoid fracture type A according to Eysel and Roosen. Based on the minor dislocation, ventral surgery was performed: Preoperative computer tomography (CT) image (**A**), intraoperative X–ray image (**B**), and postoperative CT image (**C**)

**Fig. 3:**
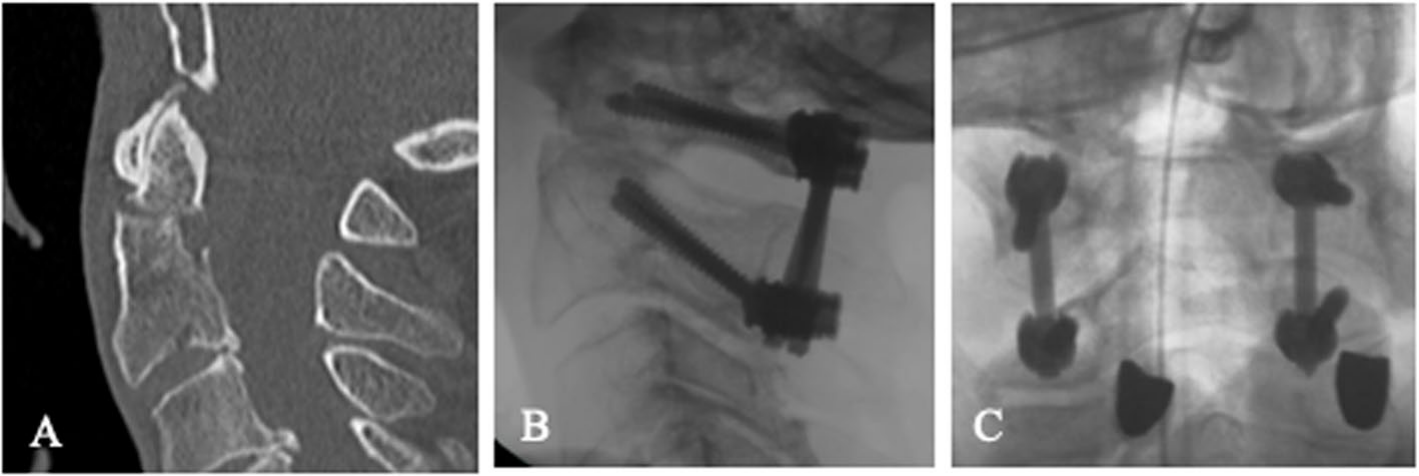
An 95–year–old male patient with odontoid fracture type A according to Eysel and Roosen: Based on the dislocation and given instability, dorsal surgery was performed: Preoperative CT image (**A**), intraoperative X–ray image (**B**, **C**)

Two different surgical procedures were used to stabilize the fracture. In both surgical procedures, the reduction was performed as a closed procedure. The ventral operative treatment was carried out via a standard Smith–Robinson approach at the upper cervical spine [21]. Two guide wires were inserted into the odontoid from ventrocaudal to craniodorsal. The guide wires were subsequently overscrewed with cannulated screws (Fig. 2A, B, C). The dorsal operative treatment was performed via a median approach. Dorsal instrumentation was performed according to Harms et al. [22] (Fig. 3A, B, C) with a Mountaineer OCT Spinal System (DePuy Spine Inc., Raynham, MA, USA).

A first control CT scan was performed before discharge from the hospital for all patients, regardless of the treatment regime, to detect possible secondary dislocation and to control implant placement in surgical cases. A second control CT scan 6 weeks after the initial trauma was recommended to detect possible secondary dislocation as well as trabecular bone fracture healing rate.

### Outcome analysis

The main endpoints of the present study were (1) the range of motion of the cervical spine at the follow–up examination and (2) the trabecular bone fracture healing rate, detected by CT scans at the follow-up examination.

A routine follow–up examination of patients took place after 6 weeks. All motions were performed in a medically controlled manner. The gross mobility of the cervical spine (extension/flexion, rotation, lateral bending) was recorded by the examining physician in all patients. The following three–step range–of–motion score (ROMS) was used to classify range of motion: range of motion was scored as “0” in patients without any mobility in the cervical spine, “1” in patients with limited mobility, and “2” in patients with almost free mobility. Although range-of-motion measurement devices (e.g. goniometer) are promising, their practicability is questionable [23]. Some degrees of exercise make no noticeable clinical difference to patients. Thus, only gross ROMS was assessed to describe the patient’s cervical spine motion.

Pain and other post-treatment complications were recorded. Routine CT imaging 6 weeks after the cervical spine injury was analyzed. Secondary dislocation, implant failure and the trabecular bone fracture healing rate was assessed. Bone healing was defined by the detection of trabecular bone across the fracture line.

### Complication analysis

For all patients, every complication was recorded and classified according to Dindo et al. [24]: Grade I is defined as any deviation from a normal treatment process, Grade II includes deviations that could be managed by additional pharmacological therapy, Grade III includes complications that had to be treated with a surgical intervention, and Grade IV describes all life–threatening complications. The death of a patient is classified as Grade V.

### Statistical data analysis

The data were tested for normality distribution with the Anderson–Darling and D’Agostino and Pearson test (age and ISAR score). If a normal distribution was found, a one–way ANOVA was performed comparing the three treatment groups. If the normality test failed, the Kruskal–Wallis test with multiple comparisons was performed. In comparing two groups, the Mann–Whitney U–test was performed if distribution was not considered normal. The Fisher´s exact test was applied to the testing of bivariate or categorical data. In all significance tests for differences and associations, a *p*–value < 0.05 was considered to indicate statistical significance. Statistical data analysis was performed using GraphPad Prism (Version 8.2.1, San Diego, CA, USA).

## Results

### Patient characteristics

In the period between January 2012 and December 2017, a total of 72 patients (40 female, 32 male) were included in this study. The mean age was 83.2 ± 7.5 years (range: 65–100 years). The mean ISAR score was 2.6 ± 0.8 (range: 2–5). Between the examined groups (conservative treatment, ventral surgery, and dorsal surgery), there was no significant differences regarding the distribution of patient age, gender or ISAR score. Therefore, the groups were categorized as comparable.

The main injury mechanism was fall from a low height (< 2 m, *n* = 68; 94.4%). Two patients (2.8%) fell from a great height (≥ 2 m). Another two patients (2.8%) were involved in a traffic accident. The mean GCS upon admission was 14.6 ± 1.3 (range: 8–15).

Concomitant injuries were present in 40 patients (55.6%). The most frequent concomitant injuries were scalp laceration (*n* = 17; 23.6%), fracture of the nasal bone (*n* = 11; 15.3%), and craniocerebral trauma (*n* = 7; 9.7%) with accompanying intracranial hemorrhage in 5 cases. In 3 patients (4.2%), an additional injury to the spine was diagnosed.

### Treatment

The number of patients in the different treatment groups is shown in Table 1. A total of 19 patients (26.4%) were treated conservatively (conservative treatment). There were no crossovers to surgical therapy. Surgical stabilization of the fracture was performed in 53 patients (73.6%). A ventral approach with screw osteosynthesis of the fractured odontoid was performed in 35 patients (48.6%; ventral surgery). In 18 patients (25.0%), surgery was performed using a dorsal approach (dorsal surgery). In two patients, a halo fixator was applied as emergency treatment until final surgical treatment could be performed.

**Table 1:** Number of patients in the different treatment groups

Odontoid Fracture Type	Conservative Treatment	Ventral Surgery	Dorsal Surgery
Type A	3 (4.2%)	10 (13.9%)	1 (1.4%)
Type B	16 (22.2%)	25 (34.7%)	17 (23.6%)

**Fig. 4:**
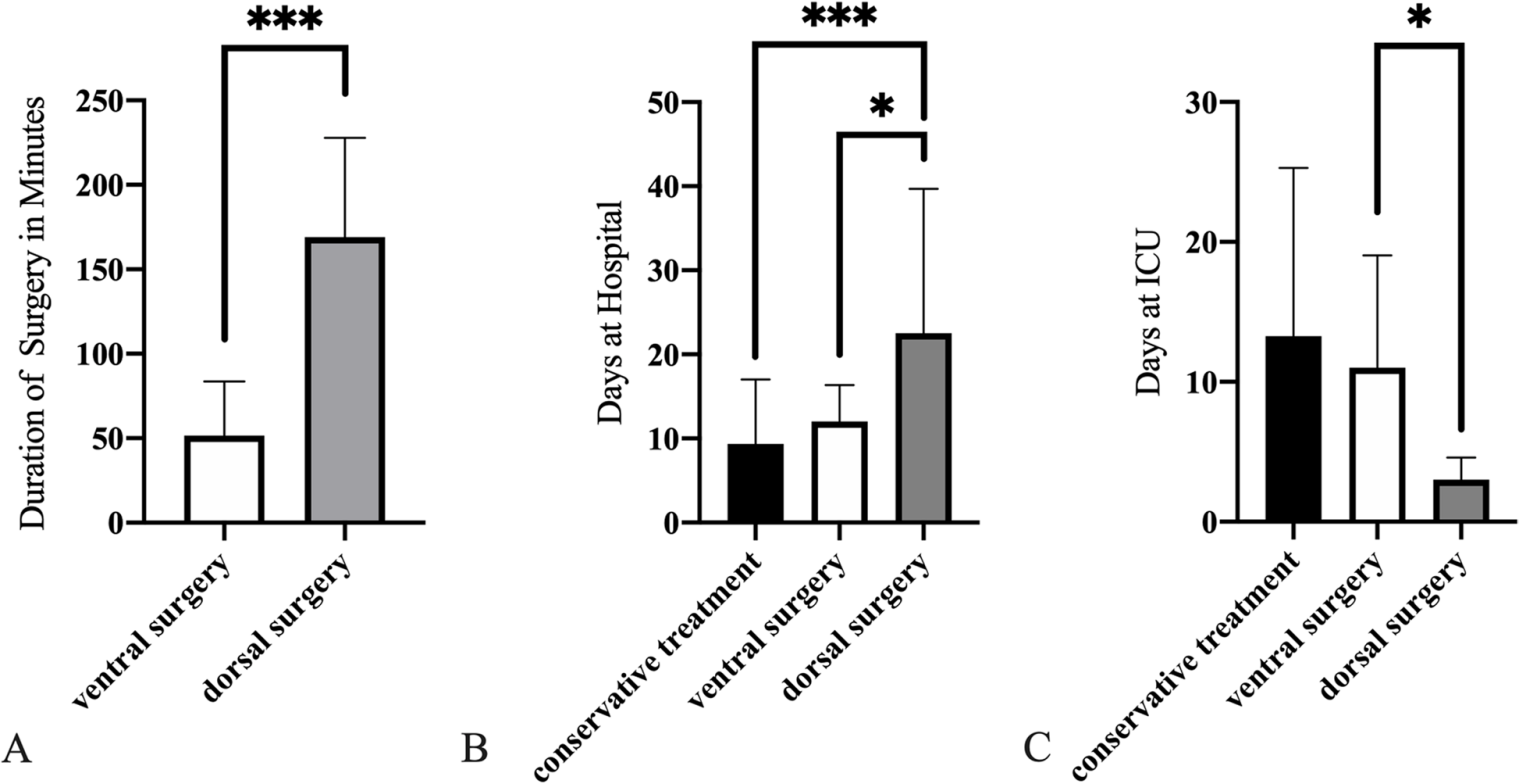
Mean duration of surgery in minutes (**A**), mean duration of treatment in hospital in days (**B**), mean duration of treatment at the intensive care unit (ICU) in days (**C**). The significances were indicated with an asterisk: * *p* < .05, *** *p* < .0001

The duration of surgery in patients from the dorsal surgery group (169.1 ± 58.7 min; range: 86–315 min) was significantly longer than in patients from the ventral surgery group (51.5 ± 32.1 min; range: 23–181 min; *p* < 0.0001; Fig. 4A, Table 2).

**Table 2:** Days at hospital and at intensive care unit (ICU) as well as duration of surgery according to the three different treatment options

	**Conservative Treatment**	**Ventral Surgery**	**Dorsal Surgery**
**Days at Hospital**			
*n* (%)	19 (26.4)	35 (48.6)	18 (25.0)
mean ± SD	9.4 ± 7.7	12.0 ± 4.3	22.5 ± 17.2
range	2–30	5–23	8–73
**Days at ICU**			
*n* (%)	4 (5.6)	6 (8.3)	5 (6.9)
mean ± SD	13.3 ± 12.0	11.0 ± 8.0	3.0 ± 1.6
range	4–30	6–23	1–5
**Duration of Surgery**			
*n* (%)		35 (48.6)	18 (25.0)
mean ± SD		51.5 ± 32.1	169.1 ± 58.7
range		23–181	86–315

Overall, the patients were hospitalized for 13.9 ± 11.0 days (range: 2–73 days). Patients from the dorsal surgery group were hospitalized for 22.5 ± 17.2 days (range: 8–73 day), which is significantly longer than patients in the ventral surgery group (12.0 ± 4.3 days; range: 5–23 days; *p* = 0.0162). However, hospitalization was shortest for the conservative treatment group (9.4 ± 7.7 days; range: 2–30 days; Fig. 4B, Table 2).

Patients from the ventral surgery group were treated in the ICU for a mean of 11.0 ± 8.0 days (*n* = 4; range: 6–23 days). Mean treatment time in the ICU for patients from the dorsal surgery group (3.0 ± 1.6 days; *n* = 5, range: 1–5 days; Fig. 4C, Table 2) was significantly shorter (*p* = 0.0444). Patients from the conservative treatment group spent, on average, 13.3 ± 12.0 days (*n* = 4; range: 4–30 days) in the ICU.

The cervical spine was immobilized in 60 patients (83.3%). Table 3 illustrates the type of immobilization and application time.

**Table 3:** Number, type, and duration of immobilization in the different treatment groups

	**Conservative Treatment**	**Ventral Surgery**	**Dorsal Surgery**
Rigid Collar	12 (16.7%)	20 (27.8%)	6 (8.3%)
Soft Collar	7 (9.7%)	11 (15.3%)	4 (5.6%)
No Collar	0 (0.0%)	4 (5.6%)	8 (11.1%)
Time with Collar (in Weeks) Mean ± SD (Range)	6.0 ± 0.0 (6–6)	4.5 ± 2.1 (0–6)	1.3 ± 1.5 (0–6)

### Functional and clinical outcome

A total of 43 patients were re–examined (follow–up rate: 59.7%). The mean follow-up time was 2.7 ± 2.1 months (range: 1–9 months). Patients from all groups were re–examined (conservative treatment: *n* = 9; ventral surgery: *n* = 22; dorsal surgery: *n* = 12). Between the follow–up groups, there were no significant differences between the age, gender and ISAR score distributions.

**Fig. 5:**
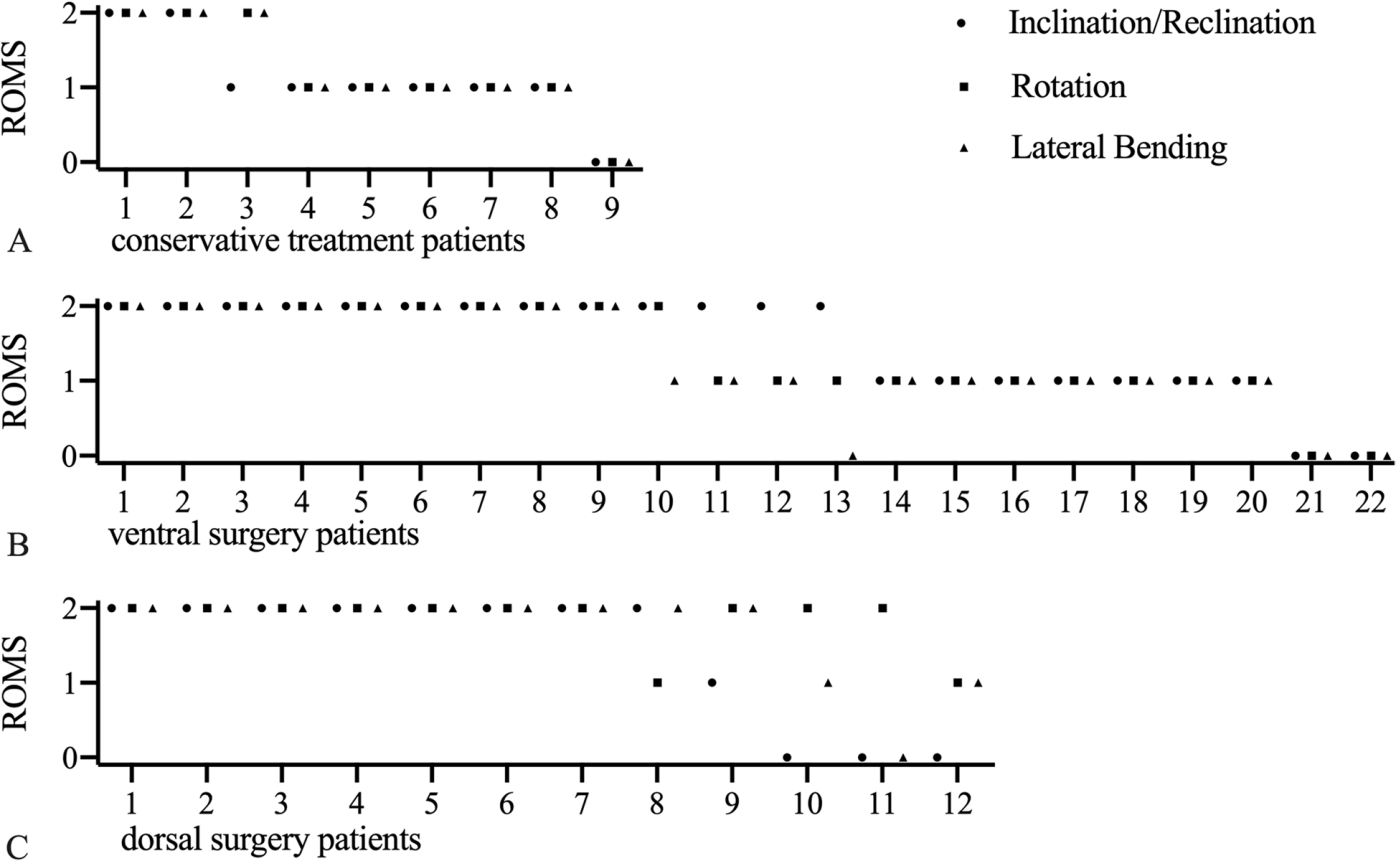
Cervical spine ROMS of all patients following conservative treatment (**A**), ventral surgery (**B**) and dorsal surgery (**C**) at the follow up

The ROMS distribution of all followed-up patients in the three treatment groups are shown in Fig. 5. Patients with a complete loss of cervical spine mobility towards all directions are rare and were only found in the conservative treatment group (Fig. 5A) as well as in the ventral surgery group (Fig. 5B). In this cohort, full cervical spine mobility was preserved best after dorsal surgery (Fig. 5C).

For reclination and inclination, the mean ROMS in the conservative treatment group was 1.1 ± 0.6. In the ventral vs. dorsal surgery group, mean ROMS for reclination and inclination was 1.5 ± 0.7 vs. 1.5 ± 0.9, respectively. Comparing the surgically and conservatively treated patients, there was no significant difference (*p* = 0.3299). There was also no significant difference when comparing the ventral and dorsal operative groups (*p* = 0.9995).

For rotation, the mean ROMS in the conservative treatment group was 1.2 ± 0.7. In the ventral vs. dorsal surgery group, mean ROMS for rotation was 1.3 ± 0.6 vs. 1.9 ± 0.3, respectively. Comparing the surgically and conservatively treated patients, there was no significant difference (*p* = 0.9148). When comparing the ventral and dorsal operative groups, there was a significant difference (*p* = 0.0267).

For lateral bending, the mean ROMS in the conservative treatment group was 1.1 ± 0.8. In the ventral vs. dorsal surgery group, mean ROMS for lateral bending was 1.3 ± 0.7 vs. 1.7 ± 0.6, respectively. Comparing the surgically and conservatively treated patients, there was no significant difference (*p* = 0.9915). There was also no significant difference when comparing the ventral and dorsal operative groups (*p* = 0.1765).

At the follow-up examination, no patient from the conservative treatment group suffered from pain. In the surgical groups, 19.0% vs. 16.7% (ventral surgery vs. dorsal surgery) of patients complained of pain. The only clinical complication that occurred during the follow-up treatment was an unsteady gait. This complication was most frequent in patients undergoing dorsal surgery, at 16.7%. This unsteady gait was less frequent in the other two groups at 11.1% (conservative treatment) and 4.8% (ventral surgery). There were no significant differences between the results for the three groups (p = 0.5444).

### Radiological outcome

The trabecular bone fracture healing rate was 78.6% of patients at follow–up. The conservative group had the lowest trabecular bone fracture healing rate (72.7%). There was no significant difference when comparing trabecular bone fracture healing rate of conservative versus operative group (*p* = 0.378, odds ratio = 2.2, CI: 0.505–10.2).

The trabecular bone fracture healing rate occurred in 79.2% of patients in the ventral surgery group. The highest rate of trabecular bone fracture healing occurred in the dorsal surgery group (94.2%). There was no significant difference when comparing ventral and dorsal surgery group with trabecular bone fracture healing rate (*p* = 0.373, odds ratio = 4.2, CI: 0.561–52.29).

Screw loosening occurred only in 2 patients from the ventral surgery group. Secondary dislocation of the fracture occurred in 6 patients (2 with conservative treatment and 4 with ventral surgery).

### Complications

**Fig. 6:**
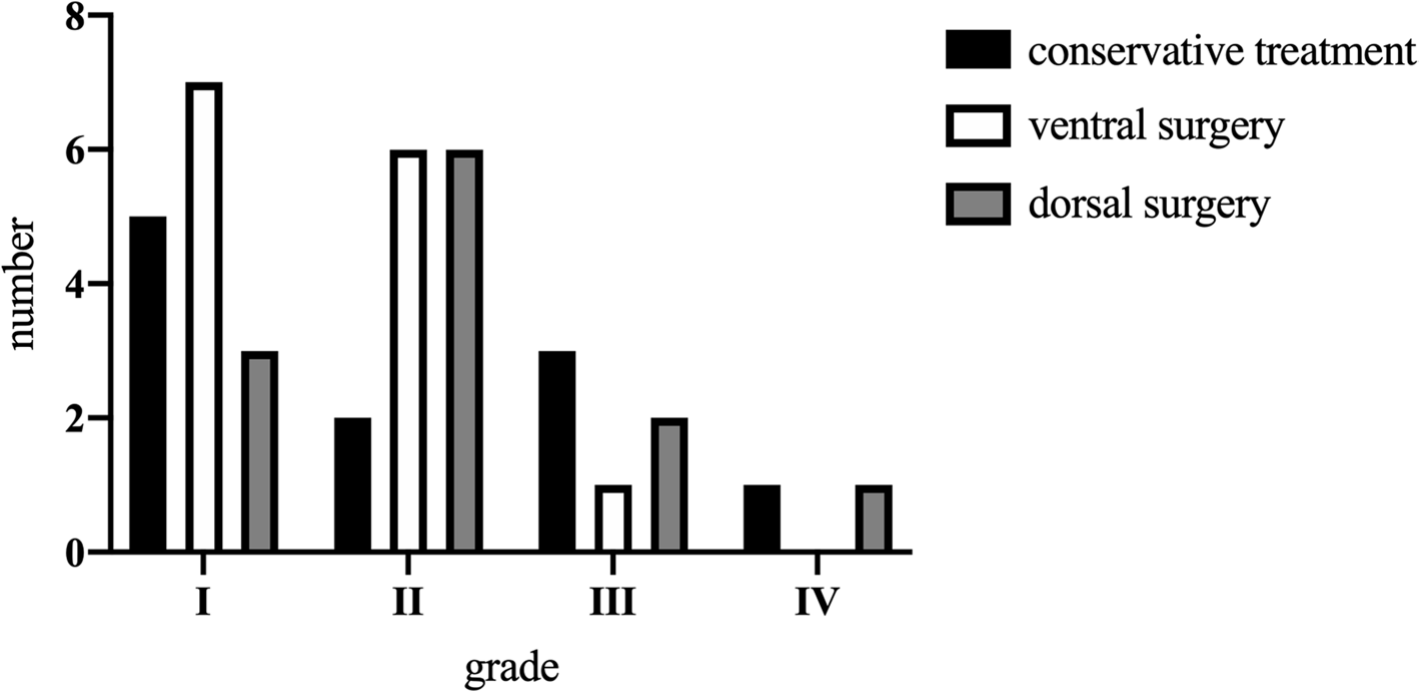
Number of complications according to grade and treatment

A total of 46 patients (63.9%) had no recorded complications. The remaining 26 patients (36.1%) had a total of 34 complications. There was no significant difference between the three treatment groups in terms of numbers and grade of complications according to Dindo et al. (Table 4; Fig. 6; conservative treatment: *n* = 8, 50.0%; ventral surgery: *n* = 13, 37.1%; dorsal surgery: *n* = 12, 66.7%). Delirium occurred most frequently in patients in the conservative treatment group (*n* = 3; 15.8%) and in patients from the dorsal surgery group (*n* = 3; 16.7%). Pneumonia occurred most frequently in patients of the ventral surgery group (*n* = 3; 8.6%). Loss of reduction occurred in patients of the ventral surgery (*n* = 4; 11.4%) and conservative treatment (*n* = 2, 10.5%) groups.

**Table 4:** Number of complications according to Dindo et al. in the different treatment groups

Grade	Complication	Conservative Treatment	Ventral Surgery	Dorsal Surgery
**I**	delirium (transient confusion)	3	1	3
	screw loosening	0	2	0
	fracture displacement	2	4	0
**II**	coprostasis	0	0	1
	hyper-/hypocalcemia	1	0	1
	hypertensive dysfunction	1	0	0
	pneumonia	0	3	1
	urinary tract infection	0	0	2
	anemia	0	1	0
**III**	wound infection	0	0	2
	gastrointestinal bleeding	0	1	0
**IV**	brain hemorrhage septic shock	1 0	0 1	1 1
**V**	death in the course of treatment	0	0	0

## Discussion

The treatment options for odontoid fracture in geriatric patients are a subject of controversy in the current literature [13, 20, 25, 26]. There are no clear instructions for further therapy, especially for type A and B fractures according to Eysel and Roosen [27–29]. These fractures could be addressed by a ventral stabilization as well as by a dorsal stabilization.

The findings of the current study confirm that the individual treatment methods have different advantages and disadvantages. For each individual geriatric patient entering the emergency room with an odontoid fracture, these treatment advantages and disadvantages should be weighed against each other, and an individual treatment plan should be constructed. The evidence from our study shows that dorsal surgical treatment may be preferable, especially in geriatric patients with a type A or B odontoid fractures according to Eysel and Roosen.

The goals in the treatment of geriatric patients differ from those of adults. The focus in geriatric patients is on maintaining independence in the activities of daily life [30]. The present study showed a trend toward dorsal surgical therapy. Patients had secure fixation with dorsal instrumentation and thus primary stability. The geriatric patients with dorsal instrumentation showed a tendency towards a better functional outcome with better mobility of the cervical spine (ROMS). This high primary stability in the fracture area allows especially the geriatric patients to achieve early autonomy [30]. Treatment by dorsal instrumentation involves fixation of the upper cervical spine and is safe and stable [20, 31, 32], especially in comparison to ventral screw osteosynthesis, which is susceptible to screw dislocation [33], and in comparison to conservative treatment, in which external fixation creates only minimal immobilization, and secondary dislocation of the odontoid fracture often occurs [12].

Especially in geriatric patients, the mobility of the cervical spine during the follow-up examination is another clinically relevant parameter and a main endpoint of the study when evaluating the different treatment methods. Most studies describe an advantage of mobility in ventral screw osteosynthesis since no mobile segments of the upper cervical spine are fixed [26, 34]. For patients with dorsal instrumentation, a considerable restriction of rotation is often described [35]. However, in the current study, rotation was less restricted following dorsal instrumentation. No significant differences were found towards inclination, reclination and lateral bending. The good mobility of the cervical spine from the dorsal surgery group patients was most likely due to non-executed immobilization. Patients from the ventral surgery and conservative treatment groups were immobilized in the cervical spine for up to 6 weeks. Patients from the dorsal group were able to move the cervical spine directly and without immobilization and could thus be physiotherapeutically exercised. Whether this rotation in the three groups is equalized in the long term must be analyzed in further studies.The complication rate of dorsal surgery patients was not significantly higher compared to the other groups, despite the invasiveness of the surgical approach. Even the length of stay in the ICU was significantly shorter than ventral surgery patients. Postoperative unsteady gait was not significantly increased. Postoperative pain was described equally in both surgically treated groups, most likely due to the short follow-up phase. Post-operative pain after cervical spine surgery only decreases noticeably after 3 to 12 months [36]. In a systematic review of a total of 1233 geriatric patients with type II odontoid fracture according to Anderson and D’Alonzo, Schröder et al. found no significant difference in the number of complications between surgical and conservative treatment or between the various surgical treatment options [37]. White et al. reported similar results regarding complications in a systematic review of the literature [38].

A disadvantage for patients in the dorsal surgery group is that the duration of surgery was significantly longer. However, the shorter length of stay of the geriatric patients at the ICU compensated for the longer duration of surgery. Patients from the conservative treatment group and ventral surgery group were treated in the ICU due to respiratory and swallowing problems.

Overall, the long hospitalization time of geriatric patients with high ISAR score is due to early complex rehabilitation already starting in the hospital. The longer hospitalization time of patients from the dorsal surgery group is most likely due to extended clinical control and wound healing. Experience shows that wounds from dorsal cervical spine surgery often shows prolonged wound healing. Furthermore, wound infections occur more frequently [5]. This was also shown in our study.

## Limitations

The present study is limited by the retrospective monocentric study design. The decision on the allocation to one of the treatment paths was based on the current recommendations but was also influenced by the patient`s medical condition. Thus, a biased choice of treatment options could not be excluded. Further studies should investigate whether patients with a lower level of dislocation also benefit from dorsal surgery. In addition, only a small group of patients was examined, which limits the significance of the present work.

Some parameters such as the intraoperative blood loss were not documented in a standardized manner at the time of the study and could thus not be analyzed. According to the literature, the follow-up period of almost 3 months is only suitable to make a first statement about a possible trabecular bone fracture healing in the geriatric population [39]. Furthermore, it should be noted that in the present geriatric patient population with a mean age of 83 years and an ISAR score of 2.6, long-term outcome is not expected [40]. Especially since these patients have a high risk of death [41].

Apart from these limitations, the data of the present study suggests that geriatric patients with a type A or B odontoid fracture according to Eysel and Roosen may benefit from a surgical treatment with a dorsal instrumentation. This finding is supported by other recent studies [20, 42]. However, all treatment options have to be weighed up against each other in every individual patient since each of the different treatment option has special disadvantages.

## Conclusions

In summary, geriatric patients with odontoid fracture type A and B according to Eysel and Roosen represent a major challenge for hospitals and the healthcare system. The treatment and surgical therapy should be chosen on an individual basis according to the patient’s condition and the fracture morphology. The findings show that the range of motion, the complications and the trabecular bone healing rate do not correlate with the different treatment strategies (conservative treatment, ventral surgery, and dorsal surgery). Altogether, dorsal instrumentation may have advantages over conservative therapy and ventral screw osteosynthesis.

## Data Availability

The datasets used and/or analyzed during the current study are available from the corresponding author on reasonable request.

## Abbreviations

CT: Computer tomography
GCS: Glasgow Coma Scale
ICU: Intensive care unit
ISAR: Identification of seniors at risk
ROMS: Range of motion score
